# Up-regulation of miR-133a-3p promotes ovary insulin resistance on granulosa cells of obese PCOS patients via inhibiting PI3K/AKT signaling

**DOI:** 10.1186/s12905-022-01994-6

**Published:** 2022-10-08

**Authors:** Xiaoman Yang, Kehua Wang, Jiajia Lang, Danyang Guo, Haixia Gao, Yue Qiu, Xiaohan Jin, Mingyue Zhang, Jiaxiu Shi, QianQian Ma, Qian Ma, Zixi Wen

**Affiliations:** 1 Shandong University of Traditional Chinese Medicine, Jinan, China; 2 Integrative Medicine Center for Reproductive and Heredity, The Affiliated Hospital of Shandong University of Traditional Chinese Medicine, No. 42 Wenhuaxi Road, Jinan, China

**Keywords:** Polycystic ovarian syndrome, Insulin resistance, Obesity, Granulosa cells, miR-133a-3p, PI3K/AKT signaling

## Abstract

**Background:**

MicroRNAs are a type of non-coding single-stranded RNA, which is involved in the regulation of ovary insulin resistance (IR). This study aims to explore the underlying mechanisms of miR-133a-3p regulating ovary IR in obese polycystic ovary syndrome (PCOS).

**Methods:**

Granulosa cells (GCs) were extracted from follicular fluids of PCOS patients (obese PCOS group and non-obese PCOS group) and healthy women (control group). The expression of miR-133a-3p in GCs was detected by qRT-PCR. The targets and pathways of miR-133a-3p were predicted by bioinformatics analyses. The protein levels of PI3K, p-AKT, GLUT4, p-GSK-3β, and p-FOXO1 were measured by Western blotting.

**Results:**

MiR-133a-3p was highly expressed in GCs from PCOS patients, especially in obese PCOS patients. The protein levels of PI3K and p-AKT was downregulated in GCs from PCOS patients. There were 11 target genes of miR-133a-3p enriching in PI3K/AKT signaling pathway. miR-133a-3p mimic downregulated the expression of PI3K, p-AKT, and GLUT4, and upregulated the protein levels of p-GSK-3β and p-FOXO1. miR-133a-3p inhibitor presented the opposite effect of miR-133a-3p mimic.

**Conclusion:**

MiR-133a-3p promotes ovary IR on GCs of obese PCOS patients via inhibiting PI3K/AKT signaling pathway. This study lays a foundation for further research on the mechanism of ovary IR in obese PCOS patients.

## Introduction

Polycystic ovary syndrome (PCOS), a kind of endocrine disease among reproductive age women and the most common reason of anovulation infertility, is complex with reproductive and metabolic features [1]. Ovary insulin resistance (IR) is considered as a key clinical characteristic of PCOS [2], especially in obese PCOS. Recent studies have found that there are impaired insulin pathways in the ovaries of PCOS patients [3]. The abnormal insulin signaling pathway has a crucial impact on the endocrine function of the ovary. Studies have shown that after 3 months of metformin treatment, the sparse menstrual manifestations of PCOS patients recover, and the average level of serum free testosterone also decreases significantly [4]. Insulin is also associated with ovulation and oocyte quality. Researches have shown that the PI3K/AKT/GLUT4 signaling in the ovary of PCOS patients is inhibited, resulting in impaired energy supply from GCs to oocytes, which leads a decrease in the rate of high-quality embryos [5]. A major challenge for poor reproductive prognosis of obese PCOS is due to incomplete understanding of the ovary IR. Thus, there is an immediate need to understand more detailed molecular mechanism underlying ovary IR so that a novel treatment strategy can be developed.

MicroRNAs (miRNAs) are a type of non-coding single-stranded RNA, which can regulate gene expression after transcription and perform their function by mediating translation suppression or directing target mRNA cleavage [6, 7]. miRNAs participate in many important processes such as growth, development, proliferation and apoptosis [8]. miRNAs have been regarded as crux blockers that control many pathways, including the insulin signaling. miRNAs can regulate insulin target organs insulin sensitivity, and thus participate in the development of IR [9–11]. However, far too little attention has been paid to the mechanism of miRNA expression and ovary IR. Our previous microarray results suggested that miR-133a-3p was differently expressed in ovarian GCs in obese PCOS patients. It indicates that miR-133a-3p may participate in the local pathological process of PCOS ovary. miR-133a is a muscle-specific miRNA that is highly conserved in the musculature of fruit flies, mice and humans and is located on three distinct chromosomes: 6p12.2, 18q11.2, and 20q13.33 [12]. Recent researchers have found that miR-133a has a close correlation with glucose metabolism. miR-133a expression in skeletal muscle decreased fivefold in T2D. Other studies showed that the expression level of miR-133a was closely related to FPG, 2 h glucose tolerance, HbA1c, and HOMA1 [13]. A study to identify genes and miRNAs involved in goat follicular development showed that the expression level of miR-133a-3p is up-regulated, which may affect goat follicular development [14]. miR-133a-3p is involved in many important pathological changes of obese PCOS like glucose metabolism disorders and follicular development disorders. These previous results give us confidence to further research the function of miR-133a-3p on ovary IR in GCs of obese PCOS patients.

We did target prediction and enrichment analysis on miR-133a-3p, and the results displayed that the target genes can be enriched in the PI3K/AKT signaling. PI3K/AKT is the main downstream molecular pathway of insulin signaling. PI3K/AKT signaling pathway-mediated insulin signaling pathways is of vital importance in regulating metabolic abnormalities and reproductive dysfunction in PCOS [15, 16]. Previous research has established PI3K/AKT signaling is inhibited in PCOS GCs, which mediates ovary IR [17]. Therefore, it is reasonable to speculate that the abnormally expressed miR-133a-3p of ovarian GCs in obese PCOS patients participates in the occurrence of glucose metabolism disorders through PI3K/AKT signaling. The objective of this study was to show the differential expressions of miR-133a-3p in the GCs of obese and normal weight PCOS patients, and their potential mechanism on ovary IR via PI3K/AKT signaling. Understanding the molecular mechanism of local ovary IR in obese PCOS patients can help provide patients with better clinical treatment.

## Materials and methods

### Cell specimens

All the GCs samples were collected from infertile patients treated with in vitro fertilization (IVF) or intracytoplasmic sperm injection (ICSI) treatment at the Center for Reproductive Medicine, the Affiliated Hospital of Shandong University of Traditional Chinese Medicine from November 2020 to June 2021. A total of 23 PCOS patients were selected as PCOS group and 10 healthy women who were infertile because of fallopian tube factor or male factor were selected as the control group. According to the body mass index (BMI), PCOS group was divided into two sub-groups, with 11 cases (BMI ≥ 24) in the obese PCOS group and 12 cases (18.5 ≤ BMI < 24) in the non-obese PCOS group. The inclusion criteria for the study group were: patients with polycystic ovary syndrome confirmed by Revised Rotterdam Diagnostic standard. Revised Rotterdam Diagnostic Criteria [18] require the presence of at least two of the following criteria: (1) Rare ovulation or anovulation; (2) Hyperandrogenism or clinical features of hyperandrogenemia; (3) Polycystic ovary. The entry criteria for the control group were: regular menstrual cycle (21–35 days), normal endocrine, and normal ovarian morphology. The age group chosen for this study was from 24 to 35 years old. The exclusion criteria for both groups were: patients with previous history of gynecological surgery; patients with abnormal chromosome karyotypes; patients with recurrent abortion and pregnancy loss; patients with sexually transmitted diseases; patients with infectious diseases; patients with reproductive system organic diseases or tumors. All subjects were fasting for 8–12 h in the early follicular phase (3–5 days after menstruation) or in the amenorrhea phase (endometrium ≤ 6 mm and all follicles ≤ 8 mm under B-ultrasound), and the fasting venous blood was taken after 30 min rest the next morning, the sex hormone, FPG and FINS were measured. The IR index (HOMA-IR) was assessed by the steady-state pattern assessment (Homa), using HOMA-IR = (FINS μIU/mL × FGP mmol/L)/22.5. After ovarian ultrasound and serum estradiol test both detected that the follicles were fully developed, human chorionic gonadotropin was administered to trigger ovulation. The eggs were retrieved under ultrasound guidance after 36 h. Place the processed oocyte and sperm in the same petri dish for co-cultivation or inject sperm into oocyte. The fertilization can be observed under a microscope. The embryos are evaluated 2–3 days after fertilization and high-quality embryos are selected for transfer or frozen embryos for selective transfer. Record the number of eggs > 14 mm on HCG day, obtained eggs, MII eggs, 2PN, 2PN cleavage and high-quality embryos, and calculate the egg capture rate, MII egg rate, normal fertilization rate, 2PN cleavage rate, high-quality embryo rate.

### Purification and culture of the GCs

The follicular fluid was pooled and centrifuged at 3000 rpm for 10 min. Then the pellets were resuspended in phosphate-buffered saline (Servicebio, Wuhan), and the suspension was slowly and carefully transferred to the same amount of FICOLL separation liquid surface and centrifuged for 5 min at 2500 rpm. After the delamination was obvious, the middle GCs layer was carefully sucked out and washed once with Dulbecco’s modified Eagle’s medium/nutrient mixture F-12 (DMEM-F12). All the GCs were cultured in DMEM-F12 (meilunbio, Dalian) containing 10% fetal bovine serum (Every Green, Zhejiang) and 1% penicillin–streptomycin-neomycin (Servicebio, Wuhan). The cells were placed in an incubator containing 5% CO_2_ at 37 °C.

### Cell transfection

The chemically synthesized miR-133a-3p mimic, miR-133a-3p inhibitor, and negative controls (NC) were used to transfect (Shanghai GenePharma) GCs. GCs were inoculated in 6-well plates. The cells were transfected using Lipofectamine 3000 (Thermofisher, USA) after growing at a 70–80% confluence.

### Quantitative real-time PCR

Total RNA from human GCs was extracted following the steps in the miRNAeasy kit (QIAGEN, Germany) instructions. We used an ultraviolet spectrophotometer (Thermo NanoDrop 2000, USA) to determine the RNA concentration and purity, and used miRNA reverse transcription kit (TAKARA, Japan) to realize reverse transcription of the total RNA into cDNA. All the cDNA samples were configured with Real-time PCR reaction system for Real-time PCR reaction. The internal standard and target miRNA primer sequence were provided and synthesized by TAKARA. The relative expression of miRNA was calculated by using 2^−ΔΔCt^. The following primers were used in the present study: miR-133a-3p: 5′-CCTCCCCTTGAACCAGCTGAAA-3′; U6-F: 5′-TGGAACGCTTCACGAATTTGCG-3′ and U6-R: 5′-GGAACGATACAGAGAAGATTAGC-3′.

### Western blot

We extracted the total proteins of GCs using RIPA lysis buffer (Beyotime Biotechnology, Shanghai), and determined the protein concentration by BCA protein concentration assay (Beyotime Biotechnology, Shanghai). The proteins (30 µg) of each group were separated by 8% SDS-PAGE and transferred to a PVDF membrane (Millipore, USA). The membrane was blocked with BSA for 1 h and combined with the specific primary antibody at 4 °C overnight: PI3K (Cell Signaling Technology, USA), phosphorylated (P-) AKT (Cell Signaling Technology, USA), GLUT4 (Affintiy, Jiangsu), p-GSK-3β (Cell Signaling Technology, USA), p-foxo1 (cell signaling technology, USA), and β-actin (proteintech, Wuhan). Then, the membrane was incubated with the corresponding horseradish peroxidase binding secondary antibody (proteintech, Wuhan) at 37 °C for 1.5 h. Western blots of proteins were visualized using enhanced chemiluminescence reagent (Beyotime Biotechnology, Shanghai), and quantified using image J.

### Statistical analysis

Clinical data analysis was performed using SPSS 26.0 software. After the normality test, the general information and hormone indicators were all in normal distribution, and were described by the mean ± standard deviation. So one-way analysis of variance was used for the comparison of general information and hormone indicators. The comparison of the rates of ovulation induction outcomes was performed using the χ^2^ test. Western Blot results were analyzed using Image J. Graphpad Prism 9 software was used for graphing and data analysis, and common one-way ANOVA-multiple comparisons were used for the comparison between groups. *P* < 0.05 indicated that the difference was statistically significant. All experiments were repeated 3 times or more.

## Results

### Characteristics of the study subjects

#### General information and hormone indicators

Table 1 summarizes general information and hormone indicators. The results showed that BMI, WHR, FINS and HOMA-IR were higher in obese PCOS group than in non-obese PCOS group and control group (*P* < 0.05). The FPG was higher in obese PCOS group than that of non-obese PCOS group (*P* < 0.05).

**Table 1:** Anthropometric and metabolic parameters of all participants

Characteristics	Obese PCOS group (*n* = 11)	Non-obese PCOS group (*n* = 12)	Control group (*n *= 10)	*P* value		
				*P*1	*P*2	*P*3
Age (years)	30.00 ± 2.83	29.73 ± 2.53	30.33 ± 2.52	0.821	0.852	0.730
BMI (kg/m^2^)	28.95 ± 4.15	20.60 ± 1.65	21.63 ± 0.92	0.000**	0.000**	0.460
WHR	0.88 ± 0.07	0.78 ± 0.07	0.77 ± 0.05	0.009**	0.022*	0.801
Years of infertility (years)	3.00 ± 1.69	2.61 ± 0.93	2.25 ± 0.50	0.527	0.336	0.634
FSH (mIU/ml)	6.00 ± 1.14	6.17 ± 1.04	6.04 ± 0.77	0.719	0.935	0.828
LH (mIU/ml)	7.11 ± 3.03	8.52 ± 4.22	4.40 ± 1.48	0.390	0.143	0.028*
E2 (pg/ml)	41.31 ± 17.03	44.26 ± 15.52	46.20 ± 7.76	0.679	0.556	0.825
P (ng/ml)	0.49 ± 0.36	0.60 ± 0.28	0.69 ± 0.25	0.469	0.440	0.738
T (ng/ml)	0.59 ± 0.08	0.51 ± 0.12	0.43 ± 0.07	0.116	0.017*	0.234
PRL (pg/ml)	14.88 ± 5.79	11.26 ± 5.50	10.96 ± 4.50	0.191	0.250	0.930
FPG (mmol/L)	5.71 ± 0.25	5.10 ± 0.36	5.38 ± 0.28	0.017*	0.198	0.246
FINS (mmol/L)	18.45 ± 5.15	9.66 ± 3.17	6.74 ± 0.61	0.015*	0.003**	0.408
HOMA-IR	4.02 ± 0.95	1.84 ± 0.77	1.65 ± 0.28	0.021*	0.010*	0.778

Data are presented as mean ± SD

*BMI*, body mass index; *WHR*, waist-to-hipratio; *FSH*, follicular stimulating hormone; *LH*, luteinizing hormone; *E*_*2*_, estradiol; *P*, progesterone; *T*, testosterone; *PRL*, prolactin; *FPG*, fasting plasma glucose; *FINS*, fasting plasma insulin; *HOMA-IR*, homeostasis model assessment of insulin resistance; *P1*, obese PCOS group versus non-obese PCOS group; *P2*, obese PCOS group versus control group; *P3*, non-obese PCOS group versus control group

*There is a significant difference between the two groups (*P* < 0.05); **Difference is highly significant (*P* < 0.01)

#### Laboratory indicators

Table 2 summarizes laboratory indicators. The results showed that the egg capture rate was lower in obese PCOS group than in non-obese PCOS group and control group (*P* < 0.05). MII egg rate and high quality embryo rate were lower in obese PCOS group than those of control group (*P* < 0.05).

**Table 2:** laboratory parameters of all participants

Characteristics	Obese PCOS group (*n* = 11)	Non-obese PCOS group (*n* = 12)	Control group (*n* = 10)	χ^2^ value	*P* value
Egg capture rate%	78.85^ab^	86.86	90.37	11.037	0.004
MII egg rate%	75.12^b^	81.51	87.70	7.976	0.019
Normal fertilization rate%	70.73	72.27	78.69	2.606	0.272
2PN cleavage rate%	93.10	93.60	95.83	0.818	0.664
High quality embryo rate%	16.30^b^	23.60	30.43	6.370	0.041

^a^*P* < 0.05 compared between obese PCOS group and non-obese PCOS group; ^b^*P* < 0.05 compared between obese PCOS group and control group; ^c^*P* < 0.05 compared between non-obese PCOS group and control group

### miR-133a-3p is up-regulated in ovarian GCs of obese PCOS group

**Fig. 1:**
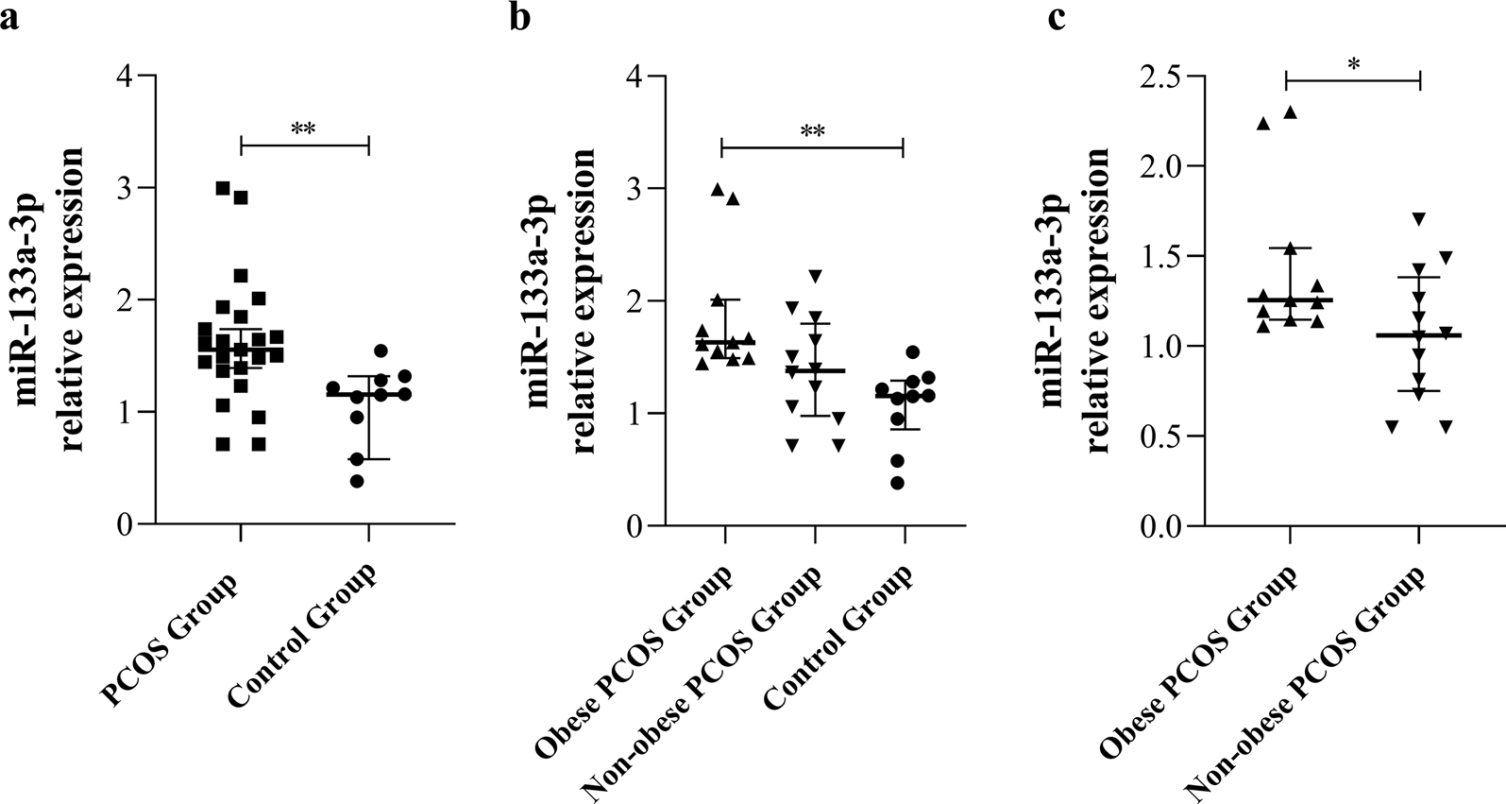
qPCR analysis validated miR-133a-3p expression in human GCs of PCOS group, obese PCOS group, non-obese PCOS group, and control group. **a** The miR-133a-3p expression in PCOS group and control group. **b** The miR-133a-3p expression in obese PCOS group, non-obese PCOS group, and control group. **c** The miR-133a-3p expression in obese PCOS group and non-obese PCOS group. Data were expressed as mean ± standard deviation (SD). *P* < 0.05

We examined the expression level of miR-133a-3p in the GCs of all participants. The results showed that the expression level of miR-133a-3p in GCs was differentially up-regulated in PCOS group than in control group (*P* < 0.05). Further multiple comparison showed that the expression level of miR-133a-3p in the GCs of obese PCOS group was significant higher than that of obese PCOS group and control group (*P* < 0.05) (Fig. 1a–c). These results suggest that miR-133a-3p is differentially expressed in ovarian GCs of PCOS patients, especially in obese PCOS patients.

### PI3K/AKT signaling is inhibited in ovarian GCs of obese PCOS group

**Fig. 2:**
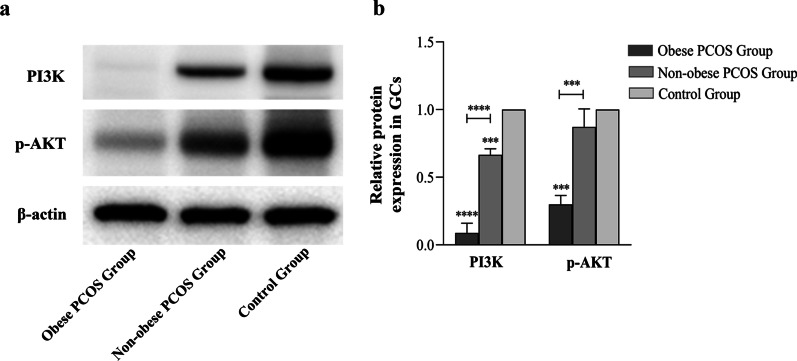
Western blotting analysis of PI3K, p-AKT in GCs of obese PCOS group, non-obese PCOS group, and control group. **a** Western blotting of PI3K and phosphor-AKT (p-AKT). **b** The relative protein levels of PI3K and p-AKT were normalized to β-actin. Data were expressed as mean ± SD. ***P < 0.001 and ****P < 0.0001

We detected the expression of PI3K, p-AKT in the GCs of obese PCOS group, non-obese PCOS group, and control group. Western blotting analysis showed that the expression level of PI3K and the phosphorylation level of AKT were decreased in obese PCOS group than those in non-obese PCOS group and control group (*P* < 0.001) (Fig. 2a, b). These results indicated that the activity of PI3K/AKT signaling in ovarian GCs of obese PCOS patients was obviously inhibited.

### miR-133a-3p inhibits PI3K/AKT signaling

**Fig. 3:**
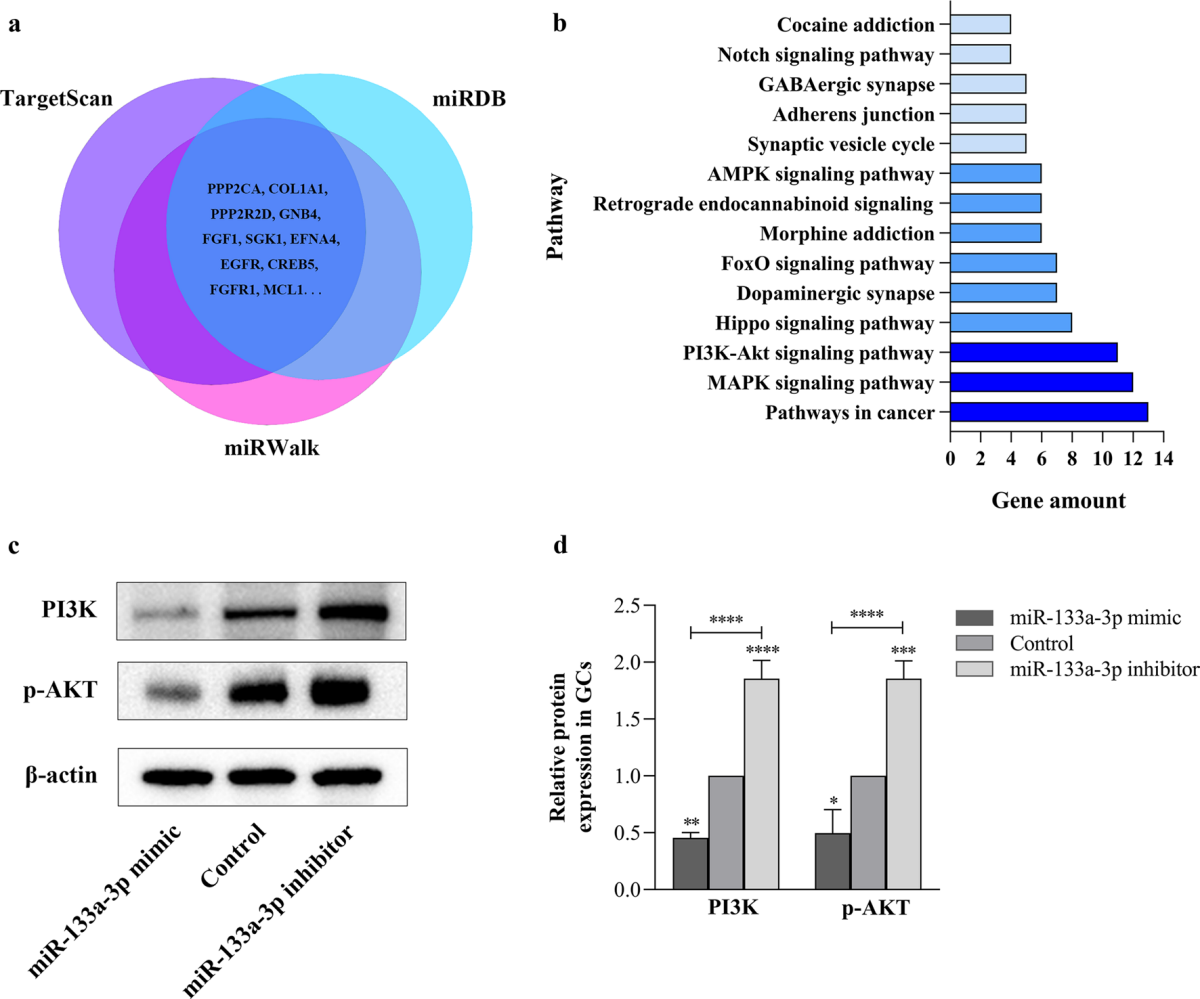
The prediction of miR-133a-3p targets and pathways. **a**–**b** The predicted miR-133a-3p targets and the signaling pathway map enriched based on the target result. **c**–**d** Western blotting analysis of PI3K and p-AKT in human GCs of miR-133a-3p mimic group, miR-133a-3p inhibitor group, and the control group. Data were expressed as mean ± SD. *P < 0.05, **P < 0.01, ***P < 0.001 and ****P < 0.0001

Select three online databases (TargetScan, miRDB, miRWalk), which contain miR-133a-3p predicted targets and experimentally verified targets (Fig. 3a). Intersect the predicted targets from the three data sets to get the final miR-133a-3p target results, a total of 321. The target results were analyzed by enrichment pathway (https://david.ncifcrf.gov/), and it was found that the targets were enriched in the PI3K/AKT signaling and participated in processes such as glucose and lipid metabolism (Fig. 3b). The prediction results show that miR-133a-3p can target 11 genes (PPP2CA, COL1A1, PPP2R2D, GNB4, FGF1, SGK1, EFNA4, EGFR, CREB5, FGFR1, MCL1) in the PI3K/AKT signaling (Fig. 3a, b). Among them, EGFR and FGFR1 have been experimentally confirmed to be the direct targets of miR-133a-3p [19].

In order to confirm that miR-133a-3p have an influence on activity of PI3K/AKT signaling in GCs, we transfected GCs of PCOS patients with miR-133a-3p mimics and inhibitors. The transfection efficiency of miR-133a-3p mimic and inhibitor was verified by PCR and the activity of PI3K/AKT signaling was detected by Western Blot. Respectively, the expression levels of PI3K and p-AKT were inhibited with miR-133a-3p overexpression, and reduced expression of miR-133a-3p resulted in the enhanced expression levels of PI3K and p-AKT (*P* < 0.05) (Fig. 3c, d), suggesting that miR-133a-3p negatively regulates PI3K/AKT signaling activity in GCs.

### *miR-133a-3p regulates ovary IR *via* PI3K/AKT signaling*

**Fig. 4:**
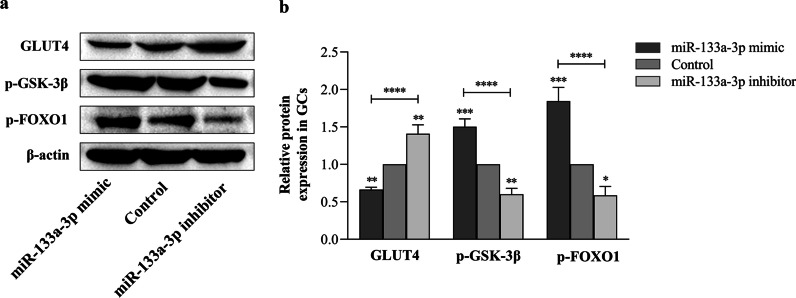
Western blotting analysis of GLUT4, p-GSK-3β, and p-FOXO1 in human GCs of miR-133a-3p mimic group, miR-133a-3p inhibitor group, and the control group. **a** Western blotting of GLUT4, p-GSK-3β, and p-FOXO1. **b** The relative protein levels of GLUT4, p-GSK-3β, and p-FOXO1 were normalized to β-actin. Data were expressed as mean ± SD. *P < 0.05, **P < 0.01, ***P < 0.001 and ****P < 0.0001

To further demonstrate that miR-133a-3p regulates PI3K/AKT signaling and affects the ovary IR of PCOS patients, we detected the protein expression of glucose metabolism-related genes downstream of PI3K/AKT signaling. Compared with control group, miR-133a-3p mimics inhibited GLUT4 protein activity and activated p-GSK-3β and p-FOXO1 protein activity (*P* < 0.01). On the contrary, miR-133a-3p inhibitors increased the expression level of GLUT4 and reduced p-GSK-3β and p-FOXO1 protein expression levels (*P* < 0.05) (Fig. 4a, b). These results show that miR-133a-3p affects PI3K/AKT signaling activity and regulates ovary IR of PCOS patients.

## Discussion

PCOS is a disease in which abnormal glucose metabolism and reproductive dysfunction coexist. Obesity is considered to be an important determinant of PCOS-related characteristics [20]. Obesity not only causes metabolic disorders, but also has a negative impact on fertility. PCOS patients are more likely to gain weight and suffer from obesity. Therefore, it is necessary to study the mechanism of PCOS based on BMI classification. We divided the study group into PCOS group with overweight or obesity and PCOS group with normal weight. We first compared the clinical and laboratory indicators of all participants in this study. The results showed that overweight or obese PCOS patients had more obvious glucose metabolism disorders and decreased oocyte quality than PCOS patients with normal weight and normal people. It suggests that PCOS combined with overweight or obesity can exacerbate the degree of IR and adversely affect the outcome of IVF. Obesity has always been considered to play a considerable role in affecting the development of glucose metabolism diseases. As a study showed, for every 1% increase in BMI, the risk of T2DM will increase by 2% accordingly [21]. A higher BMI is also associated with a worse reproductive prognosis [22]. A recent meta-analysis showed that the BMI of infertile women was significantly higher [23]. Several pieces of evidence support the negative effect of obesity on the results of assisted reproductive technology (ART). For example, obesity may prolong the time of ovulation induction, reduce the number of mature follicles and harvested eggs, and increase the cycle cancellation rate [24]. In addition, obesity may have a negative impact on the quality of oocytes and embryos. According to reports, fertilization rate, embryo transfer rate, implantation and pregnancy rate usually have a low correlation with BMI [22, 25]. The research on the pathogenesis of obese PCOS is of great significance, which helps to improve metabolic disorders and increase the success rate of ART.

A growing number of evidence showed that miRNAs contribute to the occurrence and progression of PCOS [26]. There are special miRNA differential expression profiles in serum, follicular fluid, GCs, and adipose tissue of PCOS patients [27, 28]. miRNA can participate in the occurrence of PCOS by regulating insulin sensitivity, androgen synthesis and follicle development. In this study, the expression level of miR-133a-3p in the ovarian GCs of the study group and the control group was detected, and the data analysis showed that compared with normal people, miR-133a-3p was abnormally highly expressed in the ovarian GCs of PCOS patients. Further comparison found that the expression of miR-133a-3p in obese PCOS patients was higher than that in normal weight PCOS patients. More importantly, we believe that the differential expression of miR-133a-3p in GCs may have a certain correlation with the local changes of PCOS ovaries. GCs and oocytes are closely connected, and various function changes will definitely affect the ovary microenvironment and further affect the development of oocytes. Studies have shown that miRNA can affect ovary function by acting on GCs [29]. We conducted target enrichment analysis on miR-133a-3p and found that the targets can be enriched in the PI3K/AKT signaling.

The critical importance of PI3K/AKT signaling activity in insulin action is well-established [30]. The insulin signaling pathway mediated by PI3K/AKT signaling pathway is considered to be closely related to the metabolic abnormalities and reproductive disorders of PCOS [31]. We first detected the activity of the PI3K/AKT signaling in GCs of patients in each group, and the results showed that the activity of the PI3K/AKT signaling was significantly inhibited in the GCs of obese PCOS patients. Insulin activates PI3K through insulin receptor substrate1 to promote its main downstream molecular protein AKT, which is activated by PI3K phosphorylation on ser473 and thr308. IR in PCOS leads to PI3K/AKT signaling inhibition [32]. We speculate that this inhibition state may be related to the differential expression of miR-133a-3p. Therefore, we transfected ovarian GCs with miR-133a-3p mimics and inhibitors and observed the expression changes of key proteins in the PI3K/AKT signaling, confirming the targeting effect of miR-133a-3p on the PI3K/AKT signaling. The results showed that the expression of PI3K and p-AKT in the miR-133a-3p mimic group decreased, and the expression of PI3K and p-AKT in the miR-133a-3p inhibitor group increased, which confirmed the targeted inhibition of miR-133a-3p on PI3K/AKT signaling. Several studies have already determined the negative clinical correlation between miR-133a-3p and PI3K/AKT signaling activity, like in prostate cancer [19] and nasopharyngeal carcinoma [33] tissues. These experiments can corroborate our experimental results.

In order to further explore whether the targeted inhibition of PI3K/AKT by miR-133a-3p in GCs is involved in the occurrence of local ovarian IR, we detected the expression of p-FOXO1, p-GSK-3β and GLUT4 proteins after transfection. It was found that miR-133a-3p mimics inhibited GLUT4 protein activity and activated p-GSK-3β and p-FOXO1 protein activity. On the contrary, miR-133a-3p inhibitors increased the expression level of GLUT4 and reduced p-GSK-3β and p-FOXO1 protein expression levels. They are all important proteins related to glucose metabolism disorders downstream of the PI3K/AKT signaling. Activated AKT can directly phosphorylate AS160, GSK-3β and FOXO1. AS160 can promote the transport of GLUT4 and the absorption of glucose. GSK-3β and FOXO1 can regulate gluconeogenesis and glycogen synthesis. It can be seen that miR-133a-3p mediates local IR in obese PCOS ovaries by regulating the PI3K/AKT signaling. Studies have shown that insulin promotes the decrease of glycogen synthesis in ovarian cells of PCOS patients, but the mitogenic effect of IGF-I is significantly enhanced. Therefore, ovary IR will not only change the glucose metabolism of the ovary, but also make the ovary in a hyperfunctional state, increase the responsiveness of the ovary to gonadotropins, which lead to abnormal steroidal hormone synthesis and secretion, and excessive follicle recruitment and development in the ovary [34]. In conclusion, ovary IR plays a central role in the pathogenesis of PCOS.

## Conclusion

In summary, the findings of this study demonstrate that miR-133a-3p is involved in ovary IR in granulosa cells of obese PCOS ovaries via PI3K/AKT signaling. Thus, this study contributes to our understanding of the functional role and the underlying mechanism of miR-133a-3p in ovary IR, which have important reference value for the development of drugs for ovary insulin sensitization and the expansion of clinical obesity PCOS treatment.

## Data Availability

The results and data generated during this research are all included in this article and can be obtained from the corresponding author or the first author.
